# Associations of ACE I/D polymorphism with the levels of ACE, kallikrein, angiotensin II and interleukin-6 in STEMI patients

**DOI:** 10.1038/s41598-019-56263-8

**Published:** 2019-12-23

**Authors:** Shuhong Dai, Mei Ding, Na Liang, Zhuo Li, Daqing Li, Lianyue Guan, Hongyu Liu

**Affiliations:** 1 Department of Cardiology, Zibo Central Hospital, Shandong University, No.54, Gong Qing Tuan Xi Road, Zibo, 255036 Shandong China; 2 Department of Cardiology, China-Japan Union Hospital of Jilin University, Changchun, 130033 Jilin China; 3 Office of Surgical Nursing Changchun Medical College, Changchun, Jilin China; 4 Department of Emergency, Hospital of Weifang Medical College, Weifang, Shandong China; 5 Department of Cardiology, Qilu Hospital, Shandong University, Jinan, Shandong China; 6 Department of Hepatobiliary-Pancreatic Surgery, China-Japan Union Hospital of Jilin University, Changchun, 130033 Jilin China

**Keywords:** Biological techniques, Cardiology

## Abstract

This study aimed to compare the plasma levels of angiotensin-I converting enzyme (ACE), Angiotensin II (AngII), kallikrein (KLK1) and interleukin-6 (IL-6) in ST segment elevation myocardial infarction (STEMI) patients with different ACE Insertion/deletion (I/D) polymorphisms in a Chinese population. The ACE genotypes were determined in the 199 STEMI patients and 216 control subjects. STEMI patients were divided into three groups based on the ACE genotypes. Single polymerase chain reaction (PCR) was performed to characterize ACE I/D polymorphisms. Plasma levels of ACE, AngII, KLK1 and IL-6 were measured by enzyme-linked immunosorbent assay (ELISA). We found that the DD or ID genotype was significantly independently associated with high ACE (OR = 4.697; 95% CI = 1.927–11.339), KLK1 (3.339; 1.383–8.063) and IL-6 levels (OR = 2.10; 1.025–4.327) in STEMI patients. However, there was no statistical significance between the ACE I/D polymorphism and AngII plasma levels whether in univariate or multivariate logistic regression. Additionally, we detected a significantly positive correlation between plasma KLK1 levels and IL-6 levels in STEMI patients (r = 0.584, P < 0.001). The study showed high levels of ACE, KLK1 and IL-6 were detected when the D allele was present, but AngII plasma levels was not influenced by the *ACE* I/D polymorphism.

## Introduction

Acute myocardial infarction (AMI) is a major cause of morbidity and mortality worldwide, and it has been estimated that approximately 50% to 60% of the major risk factors for CAD and AMI are determined by heritability^1^. The ST segment elevation myocardial infarction (STEMI) is one of the most severe types of AMI. It primarily occurs due to the rupture of atherosclerotic plaques. A human angiotensin I-converting enzyme (ACE) gene insertion/deletion (I/D) polymorphism, due to I/D of a 287-base pair element in intron 16 of this gene, is associated with the development of AMI by modifying ACE activity and contributing to enhanced plaque vulnerability, ulceration and thrombosis^2,3^. Our recent case-control study has also demonstrated that the ACE DD genotype is an independent risk factor for STEMI^4^.

ACE, an important common regulator of the Kallikrein-Kinin system (KKs) and the rein-angiotensin system (RAs), was revealed to convert angiotensin (AngI) into angiotensin II (AngII) and inactivate bradykinin (BK). ACE plays an important role in the physiology of blood vessels and inflammatory processes and has been widely studied to observe its correlation with various cardiovascular diseases^3^. There is substantial evidence that the ACE I/D polymorphism determines ACE, which has been demonstrated by the mean plasma ACE concentrations in individuals with the DD genotype being approximately twice that in II individuals, with ID individuals having intermediate concentrations^5^.

Among the different roles of AngII, this protein enhances the levels of cytokines, such as interleukin–6(IL-6) and IL-8, thus playing a proinflammatory role^6^. Kallikrein (KLK1), which is responsible for the generation of BK, stimulated the proinflammatory cytokines IL-6 and IL-8, as another novel action of its contribution to neovascularization^7,8^. Both inflammation and neovascularization have been reported to enhance plaque vulnerability^9^. Animal models and clinical studies demonstrated that ACE, AngII, KLK1 and IL-6 were highly expressed in unstable plaques and were significantly increased in ACS (acute coronary artery syndrome) patients^10,11^. Additionally, previous studies have shown interactions between RAs, KKs and inflammation^12,13^. In this context, we hypothesized that changes in ACE activity through the *ACE* I/D polymorphism may result in an increase in vasoactive members associated with the RAs, KKs and inflammation, such as AngII, KLK1 and IL-6. Changes in AngII, KLK1 and IL-6 levels could possibly depend on the range of ACE activity differences between the *ACE* I/D genotypes. To date, the association of ACE genetic polymorphisms with changes in ACE, AngII, KLK1 and IL-6 plasma levels has not been investigated in patients with STEMI.

The aim of the present study was to determine the possible interplay between the ACE I/D polymorphism and the important vasoactive substance of the RAs ACE, AngII, the important regulator of the KKs KLK1, and the inflammatory mediator IL-6 in patients with STEMI in the Chinese population.

## Methods

### Subjects

All procedures in studies involving human participants were performed in accordance with the ethical standards of the institutional or national research committee and with the 1964 Declaration of Helsinki and its later amendments or comparable ethical standards. This study was approved by the Ethics Committee of the Central Hospital of Zibo (approval no. ZBH05485). Informed consent for participation was obtained from all patients and their parents in an appropriate manner. A total of 260 STEMI patients admitted to the Central Hospital of Zibo from January 2014 to January 2017 and diagnosed with STEMI undergoing coronary angiography (CAG) were enrolled. All patients met the inclusion criteria, including an electrocardiography showing STEMI, detection of an increase and or decrease in troponin I with at least 1 value more than the 99th percentile upper reference limit, and at least 1 of the following: myocardial ischemic symptoms lasting longer than 30 minutes; electrocardiogram changes indicative of new ischemia (ST-segment elevation >2 mm in 2 contiguous ECG leads within 24 hours of the onset of symptoms). In addition, the CAG results for the subjects showed the presence of an acute occlusion in at least one major coronary vessel or one of its major branches. Control subjects (N = 216) had no clinical evidence of CAD, including (1) negative CAG examination results, (2) no abnormal Q wave or ST-T changes found in the resting electrocardiogram and no abnormalities found on cardiac ultrasound examinations, and (3) a negative Master exercise test. The exclusion criteria for the STEMI patients and control group were as follows: severe renal failure (serum creatinine >180 mmol/l), severe hepatic disease, peripheral angiopathy, malignant cancer and serious infections. Patients treated with ACEIs were also excluded because ACEI medication (>1 month) may affect the levels of ACE, AngII, KLK1 and IL-6^14,15^. Finally, a total of 199 patients (51 female and 148 male) with STEMI were included in this study, and 61 patients were excluded. The control subjects constituted 96 female and 120 male.

### Data collection and blood sampling

All data collection was performed with quality control. Anthropometric data on age, sex, body weight, BMI [weight (m^2^)/height (kg)], SBP, DBP, a smoking habit (one pack year: ≥20 cigarettes per day for more than 1 year), and comorbid conditions. The diagnosis of hypertension and diabetes mellitus was performed according to World Health Organization criteria A smoking habit was defined as a daily intake of >10 cigarettes continuing for more than 1 year. The other criteria are consistent with a previous study^4^. The fasting blood samples tested for routine blood, lipid profiles and coagulation tests were routinely performed in the hospital laboratory.

Fifteen milliliters of blood were taken from the arterial sheath access of all subjects during CAG prior to the injection of contrast. Blood samples were collected in tubes containing ethylene diamine tetraacetic acid (EDTA) and aprotinin and were separated by centrifugation at 4 °C for 10 minutes at 3000 rpm within one hour after collection. Then, the plasma used to measure ACE, AngII, KLK1, and IL-6 levels and leukocytes used for DNA extraction from arterial blood (during CAG). These were separated and stored at −80 °C until assayed.

### Coronary angiography (CAG)

The standard Judkins technique was implemented for coronary angiography (CAG). CAG was performed on the basis of standards of clinical practice using contrast agents (iodine amine (370)). Then, we examined the left main trunk (LM), the left anterior descending artery (LAD), the left circumflex artery (LCX), and the right coronary artery (RCA) and evaluated the stenosis of coronary arteries. Two independent and experienced interventional cardiologists blinded to the clinical information for the subjects performed the angiographic assessment.

### Measurements of ACE, AngII, KLK1 and IL-6

Plasma levels of ACE, AngII, KLK1 and IL-6 were measured by enzyme-linked immunosorbent assay (ELISA) using commercially available kits (Lengton Bioscience Co., Ltd., Shanghai; LOT: 201500506MY), which were based on a competitive enzyme immunoassay technique.

Human ACE, AngII, KLK1 and IL-6 levels in plasma were measured in strict accordance with the manufacturer’s instructions, as described previously^4^. Briefly, polyclonal antibodies specific for these three proteins were precoated onto 96-well plates and incubated for 24 hours at 4 °C. The plasma samples and horseradish peroxidase (HRP) antigen were added to each well. Standards, samples and avidin conjugated to horseradish peroxidase (HRP) were then added to the appropriate microplate wells. After rocking, the plates were covered with sealing film and incubated at 37 °C for 1 h. The plates were repeatedly washed 3 times with 1% BSA-PBS. Then, TMB substrate solution was added to each well and incubated at 37 °C for 10 min without light. After 10 min, the enzyme substrate reaction was terminated by the addition of a sulfuric acid solution. Subsequently, the optical density (OD) was measured with a Bio-Rad ELISA plate reader (Infinite M200Pro, TECAN) at a wavelength of 450 nm. The plasma levels of ACE, AngII, KLK1 and IL-6 in the samples were obtained by comparison with a calibrated standard curve.

### Detection of the *ACE* polymorphism

Genomic DNA was extracted from arterial blood (during CAG) using a human genome extraction kit (purchased from TIANGEN Biotechnology Co., LTD). The DNA concentration was measured at 260 nm, and quality controls were conducted. ACE I/D polymorphisms were determined by polymerase chain reaction (PCR). The primers had the following sequences: 5′-CTG GAG ACC ACT CCC ATC CTT CT-3′ (forward primer) and 5′-GAT GTG GCC ATC ACA TTC GTC AGA T-3′ (reverse primer). To eliminate the potential that the D allele of the ACE gene was overestimated due to failure to amplify the I allele, all DD genotype samples were confirmed by another pair of insertion-specific primers: forward, 5′-TCG GAC CAC AGC GCC CGC CAC TAC-3′; and reverse, 5′-TCG CCA GCC CTC CCA TGC CCA TAA-3′. The DNA was amplified for 30 cycles, including denaturation at 94 °C for 1 minute, annealing at 55 °C for 30 s and extension at 72 °C for 10 min. Then, 10 μl of the amplified product was examined by 1.5% agarose gel electrophoresis. PCR amplification produced fragments of 490 bp, indicating the I allele, and 190 bp, indicating the D allele, thus yielding genotypes II, ID and DD, respectively. An additional 335 bp product, a consequence of PCR using a pair of insertion-specific primers, also indicted the I allele.

### Statistical methods

The data were analyzed by SPSS version 19.0 (SPSS Co., Chicago, IL, USA) for Windows. Hardy-Weinberg equilibrium (HWE) for the distributions of *ACE* genotypes was performed by the chi square (χ^2^) test. The odds ratios (ORs) and corresponding 95% confidence intervals (CIs) for assessing the effect of the ACE I/D genotype distribution and allele frequencies on the levels of ACE, AngII, KLK1, and IL-6 in STEMI patients were calculated by logistic regression analysis. The Spearman rank test was used to define the correlation between them. For all the analyses, statistical significance was defined as P < 0.05.

## Results

### General characteristics and genotype distribution

As shown in Table 1, comparisons of the distributions of demographic general clinical and biochemical characteristics, genotype distribution and the estimated OR for each risk factor are listed. In the univariate logistic regression model, the ACE DD genotype, older age, a history of smoking habits and the presence of diabetes were found to be high risk factors for STEMI. The other variables including hypertension, body mass index (BMI), gender, white blood count (WBC), high-density lipoprotein (HDL) and plasma fibrinogen (FIB) were not significantly different between the two groups.

**Table 1:** Univariate and multivariate analyses of STEMI risk factors.

Parameters	STEMI	Control	Univariate	p*	Multivariate	p*
	(n = 199)	(n = 216)	OR (95% CI)		OR (95% CI)	
**ACE genotypes**						
II	65	88	1 (ref.)		1 (ref.)	
ID	89	108	1.167 (0.764–1.783)	0.474	1.167 (0.764–1.783)	0.474
DD	45	20	**3**.**654 (1**.**932–6**.**911)**	**<0**.**001**	**5**.**585 (2**.**459–12**.**685)**	<**0**.**001**
Age (year) <60	95	136	1 (ref.)		1 (ref.)	
≥60	104	80	**1**.**921 (1**.**303–2**.**832)**	**0**.**001**	0.830 (0.078–2.104)	0.159
BMI (kg/m^2^) <27	82	45	1 (ref.)			
≥27	117	172	1.645 (0.980–2.760)	0.060		
Gender-Female	51	96	1 (ref.)			
Male	148	120	0.431 (0.284–0.653)	0.071		
Hypertension-No	108	132	1 (ref.)			
Yes	91	84	1.324 (0.896–1.957)	0.159		
Diabetes-No	162	200	1 (ref.)		1 (ref.)	
Yes	37	16	**3**.**553 (1**.**838–6**.**876)**	<**0**.**001**	2.081 (0.089–8.432)	0.076
Smoking-No	79	156	1 (ref.)		1 (ref.)	
Yes	116	60	**3**.**230 (2**.**146–4**.**859)**	<**0**.**001**	**3**.**570 (2**.**079–6**.**132)**	<**0**.**001**
WBC (10^9^/l)	9.1 ± 0.1	10.2 ± 1.1	0.525 (0.111–1.022)	0.081		
HDL-c (mmol/l)	1.1 ± 0.2	1.3 ± 0.4	0.209 (0.101–0.430)	0.058		
FIB (mmol/l)	3.2 ± 0.7	3.3 ± 0.1	0.929 (0.710–1.271)	0.595		

STEMI: acute coronary syndrome, ACE: angiotensin converting enzyme, BMI: body mass index, WBC: white blood count, HDL-c: high density lipoprotein cholesterol, FIB: plasma fibrinogen, CI: confidence interval; OR: odds ratio. On the basis of logistic regression, statistical significance (p < 0.05) is shown in bold.

After adjusting for the other risk factors (age, BMI, Sex, hypertension, diabetes mellitus and smoking habits) the DD genotype was still significantly associated with STEMI, conferring a 5-fold higher risk (OR = 5.585; 95% CI: 2.459–12.685). A history of smoking habits was the only other independent risk factor in multivariate logistic regression, which was in accordance with other studies^16^.

### Association of the ACE I/D Polymorphism with the plasma Levels of ACE, KLK1, AngII, IL-6 in STEMI patients

To further evaluate the etiologic effects of ACE I/D polymorphisms in STEMI, we evaluated the possible influence of ACE I/D polymorphisms on STEMI-relevant traditional risk factors, plasma ACE, AngII, KLK1, and IL-6 levels. For further statistical analysis, the above plasma indicators were divided into high vs. low levels according to the mean of the STEMI patients. As shown in Tables 2 and 3, no obvious association was observed between these clinical and biochemical characteristics (age, BMI, Gender, hypertension, diabetes, WBC, HDL-c and FIB) and the ACE I/D polymorphism. However, the ACE DD and ID genotypes were found to be statistically significant associated in STEMI patients who had smoking habits (OR: 2.635, 95% CI 1.205–5.765), high plasma ACE levels (5.222; 2.290–11.907), high KLK1 (2.847; 1.277–6.545) and high IL-6 levels (2.100; 1.025–4.327) in STEMI patients.

**Table 2:** Associations between ACE I/D polymorphisms and clinical characteristics in STEMI patients.

ACE genotypes	II (n = 65)		ID (n = 89)			DD (n = 45)		
Parameters	n		n	Univariate	p*	n	Univariate	p*
				OR (95% CI)			OR (95% CI)	
Age (year) <60	33	1 (ref.)	45			17		
≥60	32		44	0.983 (0.949–1.013)	0.232	28	1.272 (0.574–2.819)	0.553
BMI (kg/m^2^) <27	29	1 (ref.)	32			21		
≥27	36		57	1.435 (0.747–2.757)	0.279	24	0.921 (0.429–1.974)	0.832
Gender-Female	40	1 (ref.)	69			33		
Male	25		20	0.464 (0.229–0.939)	0.033	12	0.582 (0.254–1.332)	0.200
SBP (mmHg) <140	57	1 (ref.)	77			41		
≥140	8		12	1.110 (0.426–2.894)	0.830	4	0.695 (0.196–2.464)	0.573
DBP (mmHg) <90	33	1 (ref.)	50			29		
≥90	32		39	8.874 (0.263–15.787)	0.129	16	6.244 (0.674–57.842)	0.107
Hypertension-No	33	1 (ref.)	40			25		
Yes	32		49	1.263 (0.665–2.398)	0.475	20	0.569 (0.261–1.242)	0.157
Diabetes-No	52	1 (ref.)	69			41		
Yes	13		20	1.159 (0.529–2.543)	0.712	4	0.390 (0.118–1.287)	0.122
Smoking-No	40	1 (ref.)	69					
Yes	25		20	**2**.**715** (**1**.**404–5**.**250)**	**0**.**003**		**2**.**635** (**1**.**205–5**.**765)**	**0**.**015**
WBC (10^9^/l)	10.1 ± 3.3	1 (ref.)	10.3 ± 2.8	0.944 (0.850–1.049)	0.284	10.1 ± 3.1	0.899 (0.790–1.022)	0.103
HDL-c (mmol/l)	1.1 ± 0.3	1 (ref.)	1.1 ± 0.2	0.379 (0.095–1.516)	0.170	1.2 ± 0.2	1.558 (0.343–7.073)	0.566
FIB (mmol/l)	3.1 ± 0.54	1 (ref.)	3.2 ± 0.7	1.159 (0.736–1.825)	0.524	3.4 ± 1.0	1.477 (0.874–2.496)	0.145

BMI: body mass index, SBP: Systolic blood pressure DBP: Diastolic blood pressure, WBC: white blood count, HDL-c: high density lipoprotein cholesterol, FIB: plasma fibrinogen.

On the basis of logistic regression, statistical significance (p < 0.05) is shown in bold.

**Table 3:** Associations between ACE I/D polymorphisms and the studied variables in STEMI patients.

ACE genotypes	II (n = 65)		ID (n = 89)			DD (n = 45)		
Parameters	n		n	Univariate	p*	n	Univariate	p*
				OR (95% CI)			OR (95% CI)	
ACE (ng/ml) <174	47	1 (ref.)	47			15		
≥174	18		42	**2**.**333** (**1**.**177–4**.**626)**	**0**.**015**	30	**5**.**222** (**2**.**290–11**.**907)**	<**0**.**001**
KLK1 (ng/ml) < 25	36	1 (ref.)	47			14		
≥25	28		42	1.149 (0.602–2.192)	0.674	31	**2**.**847** (**1**.**277–6**.**545)**	**0**.**011**
AngII (ng/l) <186	24	1 (ref.)	53			19		
≥186	41		36	0.398 (0.206–0.768)	0.006	26	0.801 (0.368–1.742)	0.576
IL-6 (ng/l) <17	36	1 (ref.)	47			14		
≥17	28		42	1.087 (0.565–2.092)	0.803	31	**2**.**100** (**1**.**025–4**.**327)**	**0**.**048**

ACE: Angiotensin converting enzyme, KLK1: kallikrein, AngII: angiotensin II, IL-6: interleukin–6. On the basis of logistic regression, statistical significance (p < 0.05) is shown in bold.

After adjusting for other risk factors, the DD genotype was still significantly independently associated with smoking habits (OR = 1.123, 95% CI: 1.041–1.212), high ACE levels (4.697; 1.927–11.339) and high KLK1 levels (3.339; 1.383–8.063) (Table 4). Whereas, there was no statistical significance between the ACE I/D polymorphism and the AngII plasma levels whether in univariate or multivariate logistic regression.

**Table 4:** The associations of ACE I/D polymorphisms with smoking habits and the studied variables in the multivariate logistic regression models.

ACE genotypes	II (n = 65)		ID (n = 89)			DD (n = 45)		
Parameters	n		n	Multivariate	p*	n	Multivariate	p*
				OR (95% CI)			OR (95% CI)	
Smoking-No	40	1 (ref.)	33			17		
Yes	25		56	**3**.**204** (**1**.**557–6**.**595)**	**0**.**020**	28	**3**.**204** (**1**.**557–6**.**595)**	**0**.**003**
ACE (ng/ml) <174	47	1 (ref.)	47			15		
≥174	18		42	**2**.**239** (**1**.**080–4**.**639)**	**0**.**030**	30	**4**.**697** (**1**.**927–11**.**339)**	**0**.**001**
KLK1 (ng/ml) <25	36	1 (ref.)	47			14		
≥25	28		42	1.046 (0.987–1.109)	0.130	31	**3**.**339** (**1**.**383–8**.**063)**	**0**.**007**

STEMI: ST segment elevation myocardial infarction, ACE: angiotensin converting enzyme, KLK1: kallikrein, CI: confidence interval; OR: odds ratio. The significant variables (smoking, ACE, KLK1) that demonstrated an independent association with ACE I/D polymorphism after multivariate logistic regression. The significant probability (P) values < 0.05 are given in bold.

**Figure 1:**
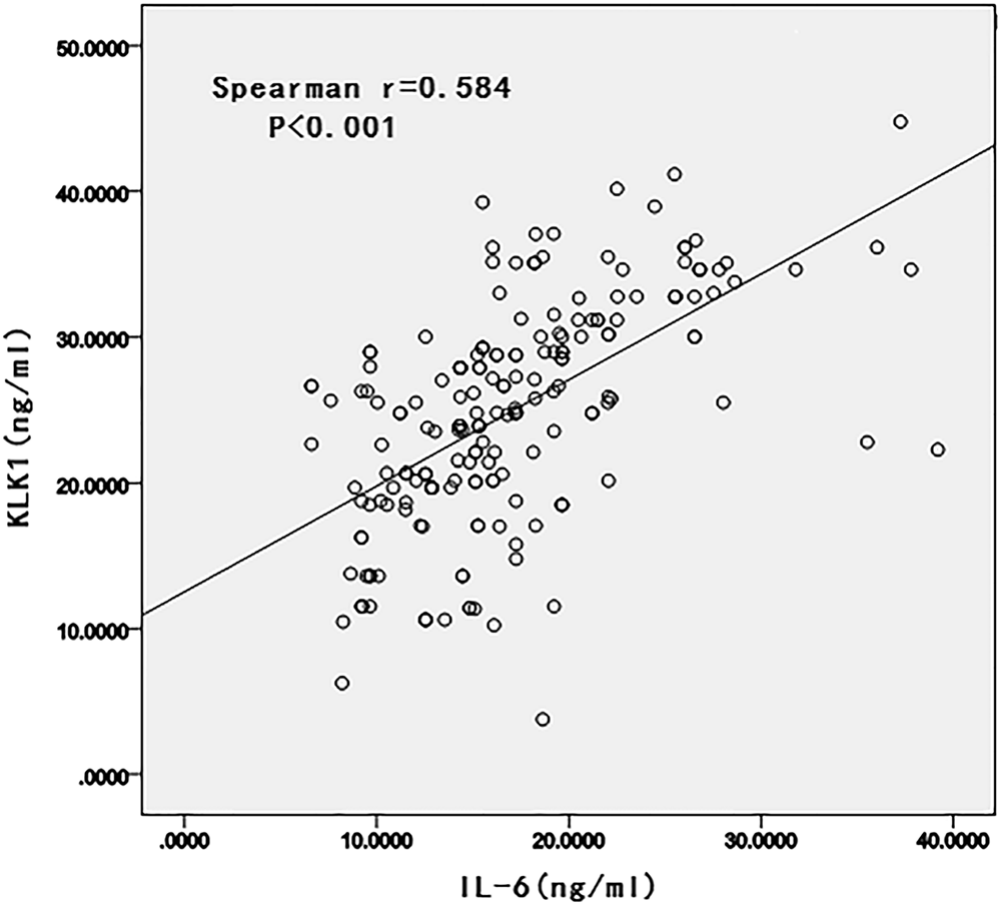
Scattered plot diagram showing the correlation between the levels of KLK1 and IL-6 among patients with STEMI.

The Spearman correlation coefficient revealed a significant positive correlation between ACE and KLK1 (r = 0.430, P = 0.038), KLK1 and AngII (r = 0.256, P < 0.001), KLK1 and IL-6 (r = 0.584, P < 0.001) in patients with STEMI (Fig. 1).

## Discussion

The ACE DD genotype is a great risk factor for AMI and has been extensively studied. There is substantial evidence showing that the ACE DD genotype may play a key role in the onset of AMI by altering plasma ACE levels or activity and increasing the instability of atherosclerotic plaques2,3. One study found that both Caucasian and Asian individuals as DD carriers conferred 1.21- and 2.47-fold risks of AMI, respectively, compared with ID or II genotype carriers, and Asian individuals as DD carriers appeared to be more prone to AMI than did Caucasian individuals3. This result was also similar to most studies in different populations or study cohorts^17,18^. One recent case-control study also demonstrated that the ACE DD genotype was an independent risk factor for AMI in a Chinese population4. A similar result was also found in the present study. After controlling for other risk factors, the DD genotype was still significantly associated with STEMI, conferring a 5-fold higher risk.

There was a significant increased risk for STEMI among individuals with DD genotypes and smoking habits. A previous study noted that The presence of the D allele also increased the risk of CAD associated with the presence of smoking habits^19^. Indeed, smoking and the D allele increase ACE expression, impair protective antioxidant mechanisms, causing endothelial dysfunction by accelerating superoxide anion formation and degradation of nitric oxide, leading to the onset of STEMI^19–21^. Our results showed that both the DD genotype and smoking habits were associated with an increased risk of STEMI in patients. Our study demonstrated that there may be an association between the DD or ID genotype and smoking in patients with STEMI (OR = 1.123, 95% CI: 1.041–1.212). As Kiyoshi Hibi *et al*. suggested that ACE gene polymorphism adds risk for the severity of coronary atherosclerosis in smokers^22^.

Previous studies have shown that subjects with the D polymorphism had a higher level of plasma ACE protein than did those with the I polymorphism. Intermediate levels were observed in heterozygotes (ID genotype)^5^. Similar results were also found in a case-control study suggesting that subjects with the DD genotype had a 2.91-fold higher risk of STEMI than those with the II genotype^23^. Similar results were also widely indicated by other studies^24^. The effects of DD genotype of the *ACE* I/D polymorphism on plasma ACE levels and activity may be an important mechanism for the association of the ACE I/D polymorphism with the most urgent STEMI events^3^. Our results suggest that STEMI patients with the *ACE* DD genotype showed the highest mean ACE plasma level, while patients with the *ACE* II genotype had the lowest mean plasma ACE level, in accordance with previous reports^5^.

Our present study revealed that the KLK1 plasma levels were higher in patients with STEMI carrying the D allele (ID/DD) compared to those lacking this allele (II). Although we have not obtained the exact mechanisms beyond this correlation, as we pointed out above and previous studies suggest that individuals with the D allele have significantly higher ACE plasma levels than do those with the I allele, which was validated by a substantial investigation^2^. The 47% phenotypic variance in ACE plasma levels was clarified by the ACE I/D gene polymorphism, and ACE plasma levels were 85% higher in DD subjects compared to II subjects, and ID subjects showed intermediate levels^5^. In addition, ACE is common to both RAS and KKS but plays different roles: it processes AngI to AngII and is the main BK-inactivating peptide^25^. KLK1 is the only member of the tissue KKS family that is responsible for the generation of BK. Subsequently, BK promotes vasodilation by the enhanced formation of nitric oxide (NO)^26^. H. Buikema *et al*. demonstrated that the arteries from DD patients with a smaller capacity for NO release in response to stimulation^27^. Furthermore, Kakoki *et al*. proposed that the D allele was associated with rapid BK degradation as a result of higher levels and activity of ACE^28^. It is tempting to speculate that the high KLK1 levels observed in patients with the D allele may play an important compensatory role in their increased BK degradation.

Urata *et al*. first showed *in vitro* two pathways for the generation of AngII in human cardiac ventricles. A serine proteinase-dependent enzyme, which was identified as a new member of the chymase family, was responsible for approximately 80% of total AngII, while ACE-dependent AngII generation only accounted for <11% of the AngII^29^. Despite the association of the D allele with increased levels of ACE, previous studies by varying degrees of ACEIs and computer simulations of the influence of genetically changing the levels of ACE revealed that such modest changes in ACE levels alter the levels of the substrate BK but had little effect on the active peptide AngII^30^. Furthermore, cellular studies stated that ACE has an even stronger affinity for BK than for AngI^31^. In the literature, we agree with the theory of Smithies that the decrease in the level of the active ACE substrate BK probably mediates the adverse effects of the D polymorphism but not the alteration in the level of AngII^32^. In the present study, we did not observe a difference in the level of active AngII regarding the ACE I/D polymorphism.

STEMI primarily occurs due to rupture of atherosclerotic plaques. In this process, inflammation is thought to play a key role along with several other risk factors^33^. Previous investigations have shown that a high level of IL-6 is an independent predictor of adverse events in patients with ACS^34^. Our data showed that the presence of the D allele is related to higher IL-6, with 18.6 ng/ml in DD and 18.0 ng/ml in ID versus 14.7 ng/ml with the II genotype^15^. As mentioned above, the results indicated that ACE DD has been reported to increase plasma ACE in accordance with previous studies. Increased ACE levels by the ACE I/D polymorphism may accelerate the expression of inflammatory cytokines, such as IL-6 and IL-8^15,35^.

In this study, we detected a significant positive correlation between plasma KLK1 levels and IL-6 levels in STEMI patients (r = 0.584, P < 0.001). Previous studies have demonstrated that most of the biological functions of KLK1 are mediated by kinin receptor signaling, which can upregulate the expression of pro-inflammatory cytokines such as IL-6 and IL-8^26^. A recent study suggests that KLK1 may also activate protease-activated receptors (PARs), leading to intracellular Ca2+ mobilization and phosphorylation of MAPK signaling and subsequent cytokine production (IL-6, CCL-2, and IL-8)^36^. Additionally, both inflammation and angiogenesis correlate with vulnerable plaques^37^. KLK1 is highly expressed in unstable plaques, implying that KLK1 contributes to angiogenesis and is involved in inflammatory activation, leading to unstable plaque formation^10^. Clinical studies have shown that both elevated plasma IL-6 and KLK1 levels may increase plaque vulnerability and are associated with the severity of STEMI^10,34^. These reports provide evidence that supports the present findings.

Controversy surrounds the effect of the ACE I/D polymorphism on CAD and AMI^38^. Most subsequent studies have indicated no significant influence of this polymorphism on the extent of CAD but mainly on the onset of AMI, especially in STEMI. These data imply a possible mechanism involving plaque vulnerability, ulceration and thrombosis^3^. In this context, it is reasonable to hypothesize that alterations in ACE activity through the ACE I/D polymorphism may result in an increase in the expression levels of IL-6 and KLK1, which increases plaque vulnerability.

### Study limitations

First, the results here are preliminary due to the limitation of the small number of patients. Second, it would be of interest in future animal and clinical studies to better confirm the mechanism of the association between the ACE I/D gene polymorphism and the level of ACE, IL-6, and KLK1, although we hypothesized that alterations in ACE activity through the ACE I/D polymorphism may result in an increase in the expression levels of these factors.

## Conclusion

To our knowledge, this study is the first to show that high levels of ACE, KLK1 and IL-6 are detected when D allele is present, but AngII was not influenced by ACE I/D polymorphism.

## Data Availability

The dataset analyzed during the current study is available from the corresponding authors on reasonable request.
